# Two new species of *Pseudocosmospora* (Hypocreales) revealed through morphological and phylogenetic analyses

**DOI:** 10.3897/mycokeys.128.180941

**Published:** 2026-02-16

**Authors:** Xiao-Qian Wu, Wen-Ying Zhuang, Zhao-Qing Zeng

**Affiliations:** 1 State Key Laboratory of Microbial Diversity and Innovative Utilization, Institute of Microbiology, Chinese Academy of Sciences, Beijing 100101, China Institute of Microbiology, Chinese Academy of Sciences Beijing China; 2 University of Chinese Academy of Sciences, Beijing 100049, China University of Chinese Academy of Sciences Beijing China

**Keywords:** Fungicolous fungi, morphology, Nectriaceae, phylogeny, taxonomy

## Abstract

*Pseudocosmospora* species typically occur on stromata of diatrypaceous and xylariaceous fungi and are mainly distributed in tropical and temperate areas. Some of them play important roles in biomedicine and agriculture due to their production of bioactive secondary metabolites. In this study, *Pseudocosmospora chlamydospora***sp. nov**. and *P. chloroxantha***sp. nov**. from Beijing, China are described based on morphological features and phylogenetic analyses of combined ITS, LSU, *tub2*, *rpb1* and *tef1-α* sequences. Detailed descriptions and illustrations of the novel taxa are provided, and their distinctions from close relatives are discussed.

## Introduction

Historically, the nectroid fungi with small, reddish, KOH+, smooth and thin-walled perithecia were placed in *Cosmospora* sensu lato (Samuels et al. 1991; Rossman et al. 1999). However, the phylogenetic analysis showed that the genus was heterogeneous (Zhang and Zhuang 2006). Efforts were made continuously towards establishment of a monophyletic *Cosmospora* Rabenh. as well as its allies (Luo and Zhuang 2008, 2010; Samuels et al. 2009; Gräfenhan et al. 2011; Schroers et al. 2011). As part of this taxonomic revision, Herrera et al. (2013) established the genus *Pseudocosmospora* C.S. Herrera & P. Chaverri, with *P. eutypellae* C.S. Herrera & P. Chaverri as the type species, to accommodate *C. vilior* (Starbäck) Rossman & Samuels and the related taxa occur on *Eutypa* Tul. & C. Tul., *Eutypella* (Nitschke) Sacc., and *Biscogniauxia* Kuntze.

Members of *Pseudocosmospora* are usually distributed in tropical and temperate regions and occur on fruitbodies of diatrypaceous and xylariaceous fungi. Some species have significant importance in agriculture and biomedicine due to their capability of producing bioactive secondary metabolites (Lee et al. 2011; Hsiao et al. 2014; Shiono et al. 2016; Nakamura et al. 2019). Morphologically, *Pseudocosmospora* species typically have solitary to gregarious, scarlet, subglobose to obpyriform, blunt-papillate perithecia that collapse laterally when dry and mostly less than 250 μm high; they possess cylindrical to narrowly clavate asci with eight ellipsoidal, 1-septate ascospores and form acremonium- to verticillium-like asexual states with cylindrical phialides that produce ellipsoidal, ovoid or reniform, aseptate ​conidia (Herrera et al. 2013; Zeng and Zhuang 2021). Currently, there are 17 globally accepted species in the genus, 12 of them have been reported in China (Herrera et al. 2013; Zeng and Zhuang 2017, 2021, 2022, 2023).

During the examinations of hypocrealean fungi collected from Beijing, China, two interesting fungicolous specimens were encountered. Morphological observations and phylogenetic analyses based on combined nuclear ribosomal DNA ITS1-5.8S-ITS2 (ITS), large subunit of nuclear ribosomal DNA (LSU), β-tubulin (*tub2*), RNA polymerase II subunit one (*rpb1*) and translation elongation factor 1-α (*tef1-α*) sequence data determined their taxonomic position in *Pseudocosmospora* and are new to the genus. Morphological differences between these novel taxa and their close relatives are compared in detail.

## Materials and methods

### Sample collection and strains isolation

Fresh specimens were collected from Baili Landscape Gallery in Yanqing District and Miyun Reservoir area in Miyun District. The collection sites were located in mixed forest vegetation. All specimens were preserved in Herbarium Mycologicum Academiae Sinicae (**HMAS**). Cultures were obtained through single ascospore isolation: fresh perithecia were surface-sterilized by immersion in 75% ethanol for 1 min, followed by three rinses with sterile water, then crushed with tweezers in 200 µL of sterile water to release the ascospores; and finally the spore suspension was spread evenly onto potato dextrose agar (**PDA**) medium. After 2–7 days of incubation, colonies were subcultured on fresh PDA. All strains were deposited in the China General Microbiological Culture Collection Center (**CGMCC**).

### Morphological characterization

The methods of Zeng and Zhuang (2022) were generally followed for morphological observations. Strains were cultured on three different media: PDA, cornmeal dextrose agar (**CMD**), and synthetic low-nutrient agar (**SNA**), and the plates were incubated under a 12/12 h light/dark photoperiod for 28 d at 25 °C. Colonies were photographed and diameters were measured. Photographs of macroscopic features were taken with a Leica DFC450 digital camera (Wetzlar, Germany) attached to a Leica M125 stereomicroscope (Milton Keynes, UK). Perithecial wall reactions were observed with 3% KOH and 100% lactic acid. Perithecia sections (ca. 6–8 μm thick) were made using a freezing microtome (YD-1508-III, Jinhua, China). The anatomic characteristics of perithecia, asci, ascospores, conidiophores, conidia and chlamydospores were examined, photographed, and measured using a Zeiss AxioCam MRc 5 digital camera (Jena, Germany) attached to a Zeiss Axio Imager A2 microscope (Göttingen, Germany).

### DNA extraction, PCR amplification, and sequencing

Genomic DNA was extracted from fresh mycelia of *Pseudocosmospora* species and the fruitbodies of their hosts using the protocol of Wang and Zhuang (2004). Five loci, namely ITS, LSU, *tub2*, *rpb1* and *tef1-α*, were amplified and sequenced with the primer pairs ITS5/ITS4 (White et al. 1990), LR0R/LR5 (Vilgalys and Hester 1990), and T1/T22 (O’Donnell and Cigelnik 1997), RPB1-Af/RPB1-Cr (Castlebury et al. 2004) and EF1-728F/EF2 (O’Donnell et al. 1998), respectively.

### Phylogenetic analyses

The sequences of *Pseudocosmospora* species along with other related species were downloaded from GenBank and the accession numbers are listed in Table 1. Sequences were assembled, aligned and trimmed by BioEdit 7.0.5 (Hall 1999), and converted using Clustal X 1.83 (Thompson et al. 1997). Phylogenetic analyses of multiple genes were conducted using the maximum likelihood (ML), Bayesian inference (BI), and maximum parsimony (MP) methods. ML analysis was determined using RAxML with the default GTRCAT model (Samatakis 2006). BI analysis was performed with MrBayes 3.1.2 using a Markov chain Monte Carlo algorithm (Ronquist and Huelsenbeck 2003). For combined sequence dataset, GTR+I+G was selected as the best-fit model using MrModeltest 2.3 (Nylander 2004), and was further used in the BI analysis. Four Markov chains were run simultaneously for 1,000,000 generations with the trees sampled every 100 generations. The first 25% of trees were set as burn-in and discarded. The remaining trees were used to calculate the Bayesian inference posterior probability (BIPP) values. The MP analysis was performed with PAUP 4.0b10, using heuristic searches with 1,000 replicates of random addition of sequences and subsequent TBR (tree bisection and reconnection) branch swapping (Swofford 2002). Topological confidence of resulted trees was tested by maximum parsimony bootstrap proportion (MPBP) with 1,000 replications, each with 10 replicates of random addition of taxa. Trees were visualized by TreeView 1.6.6 (Page 1996). Maximum likelihood bootstrap proportion (MLBP) and maximum parsimony bootstrap proportion (MPBP) ≥ 75%, and Bayesian inference posterior probability (BIPP) ≥ 0.9 are shown at the nodes.

**Table 1.** List of taxa, herbarium/strain numbers, and GenBank accession numbers used in this study.

Species	Herbarium/Strain Number	GenBank Accession Numbers					References
		ITS	LSU	*tub2*	*rpb1*	*tef1-α*	
*Corallomycetella repens*	AR 4547	JF832594	JF832679	JF832838	JF832763	JF832517	Herrera et al. 2013
*Cosmospora coccinea*	CBS 341.70^T^	MH859703	KM231692	KM232086	KM232242	KM231947	Castlebury et al. 2004; Hirooka et al. 2012; Lombard et al. 2015; Vu et al. 2019
	AR 2741	HM484537	AY489734	HM484589	AY489667	HM484515	
*C. viliuscula*	GJS 10-247	JN995629	JN939824	KC291869	KC291869	KC291843	Schoch et al. 2012; Herrera et al. 2013, 2015
	GJS 83-197	KC291732	KC291777	KC291907	KC291868	KC291849	
	GJS 86-315	KC291748	KC291779	KC291906	KC291867	KC291851	
	GJS 09-411 ^T^	JN995627	JN939826	KC291905	KC291866	KC291841	
	GJS 10-114	KJ676155	KJ676192	KJ676270	KJ676229	–	
	GJS 73-2	JN995630	JN939823	KJ676272	JQ031093	KJ676353	
*C. viridescens*	IMI 73377a^T^	KJ676171	KJ676208	KJ676292	KJ676244	KJ676373	Herrera et al. 2013, 2015
	AR 2783	KJ676142	KJ676179	KJ676253	KJ676216	KJ676336	
	CBS 102430	KJ676147	KJ676184	KJ676260	KJ676221	KJ676342	
	CBS 102433	KC291731	KC291765	KC291904	KC291865	KC291836	
*Microcera coccophila*	MAFF 241482	KC291752	KC291787	KC291936	KC291895	KC291857	Herrera et al. 2013
	GJS 83-198	KC291753	KC291778	–	KC291896	KC291850	
	GJS 98-50	KC291754	KC291784	KC291937	KC291897	KC291855	
*M. larvarum*	AR 4580	KC291751	KC291759	KC291935	KC291894	KC291832	Herrera et al. 2013
Nectriaceae sp.	MAFF 241499	KC291739	KC291789	KC291913	KC291874	KC291859	Herrera et al. 2013
Nectriaceae sp.	MAFF 241531	KC291730	KC291790	KC291918	KC291879	KC291860	Herrera et al. 2013
*P. beijingensis*	CGMCC 3.24131^T^	OP223438	OP223434	OP272862	–	–	Zeng and Zhuang 2022
*P. chlamydospora*	CGMCC 3.29438^T^	**PX630646** ^a^	**PX630644**	**PX619778**	**PX848921**	**PX848923**	This study
*P. chloroxantha*	CGMCC 3.29437^T^	**PX630647**	**PX630645**	**PX619779**	**PX848920**	**PX848922**	This study
*P. curvispora*	CGMCC 3.20176^T^	MT592897	MT592879	MT606156	MT606153	–	Zeng and Zhuang 2022
*P. eutypae*	CH 11-01^T^	KC291735	KC291766	KC291925	KC291884	KC291837	Herrera et al. 2013
	IMI 73016	KC291736	KC291786	–	KC291885	KC291856	
*P. eutypellae*	AR 4562^T^	KC291721	KC291757	KC291912	KC291871	KC291830	Herrera et al. 2013
	AR 4527	KC291720	KC291756	KC291909	KC291870	KC291829	
	GJS 10-248	KC291722	KC291772	KC291911	KC291872	KC291844	
	GJS 10-294	KC291723	KC291773	KC291910	KC291873	KC291845	
*P. henanensis*	HMAS 183528 ^T^	GU075856	GU075863	HM054103	–	HM054067	Luo and Zhuang 2010; Zhao et al. 2011
*P. hypoxylicola*	cLL 19020^T^	MN886606	MN886608	–	–	–	Lechat and Fournier 2020
*P. joca*	AR 4779^T^	KC291746	KC291762	KC291924	KC291887	–	Herrera et al. 2013
*P. metajoca*	AR 4576^T^	KC291745	KC291758	KC291923	KC291886	KC291831	Herrera et al. 2013
*P. rogersonii*	GJS 90-56^T^	KC291729	KC291780	KC291915	KC291878	KC291852	Herrera et al. 2013
	GJS 10-296	KC291727	KC291774	KC291917	KC291876	KC291846	
	GJS 09-1384	KC291726	KC291770	KC291914	KC291875	KC291840	
	GJS 10-297	KC291728	KC291775	KC291916	KC291877	KC291847	
*P. shennongjiana*	CGMCC 3.20177^T^	MT592898	MT592880	MT606157	MT606154	–	Zeng and Zhuang 2021
*P. vilior*	AR 4810^T^	KC291737	KC291763	KC291928	KC291900	KC291834	Herrera et al. 2013
	AR 4771	KC291734	KC291761	KC291926	KC291898	KC291833	Zhao et al. 2011
	PC 1246	KC291738	KC291791	KC291927	KC291899	KC291861	
	7497	HM054160	HM042407	HM054105	–	HM054069	
*Pseudocosmospora* sp.	AR 4826	KC291740	KC291764	KC291929	KC291888	KC291835	Herrera et al. 2013
*Pseudocosmospora* sp.	GJS 96-216	KC291733	KC291783	KC291930	KC291889	–	Herrera et al. 2013
*Pseudocosmospora* sp.	AR 4768	KC291724	KC291760	KC291922	KC291881	–	Herrera et al. 2013
*Pseudocosmospora* sp.	GJS 95-141	KC291749	KC291781	KC291921	KC291883	KC291853	Herrera et al. 2013
*Pseudocosmospora* sp.	CH 11-02	KC291725	KC291767	KC291919	KC291882	KC291838	Herrera et al. 2013
*Pseudocosmospora* sp.	CGMCC 3.20178	MT592899	MT592881	MT606158	MT606155	–	Zeng and Zhuang 2021
*Pseudocosmospora* sp.	GJS 95-143	KC291750	KC291782	KC291920	KC291880	KC291854	Herrera et al. 2013
*Pseudonectria pachysandricola*	AR 4592	JF832658	JF832715	JF832909	JF832791	JF832544	Hirooka et al. 2012

^T^ indicates the ex-type culture. ^a^ Numbers in bold indicate the newly provided sequences, unavailable sequences are marked with ‘–’.

## Results

### Multi-gene phylogeny

**Figure 1.**
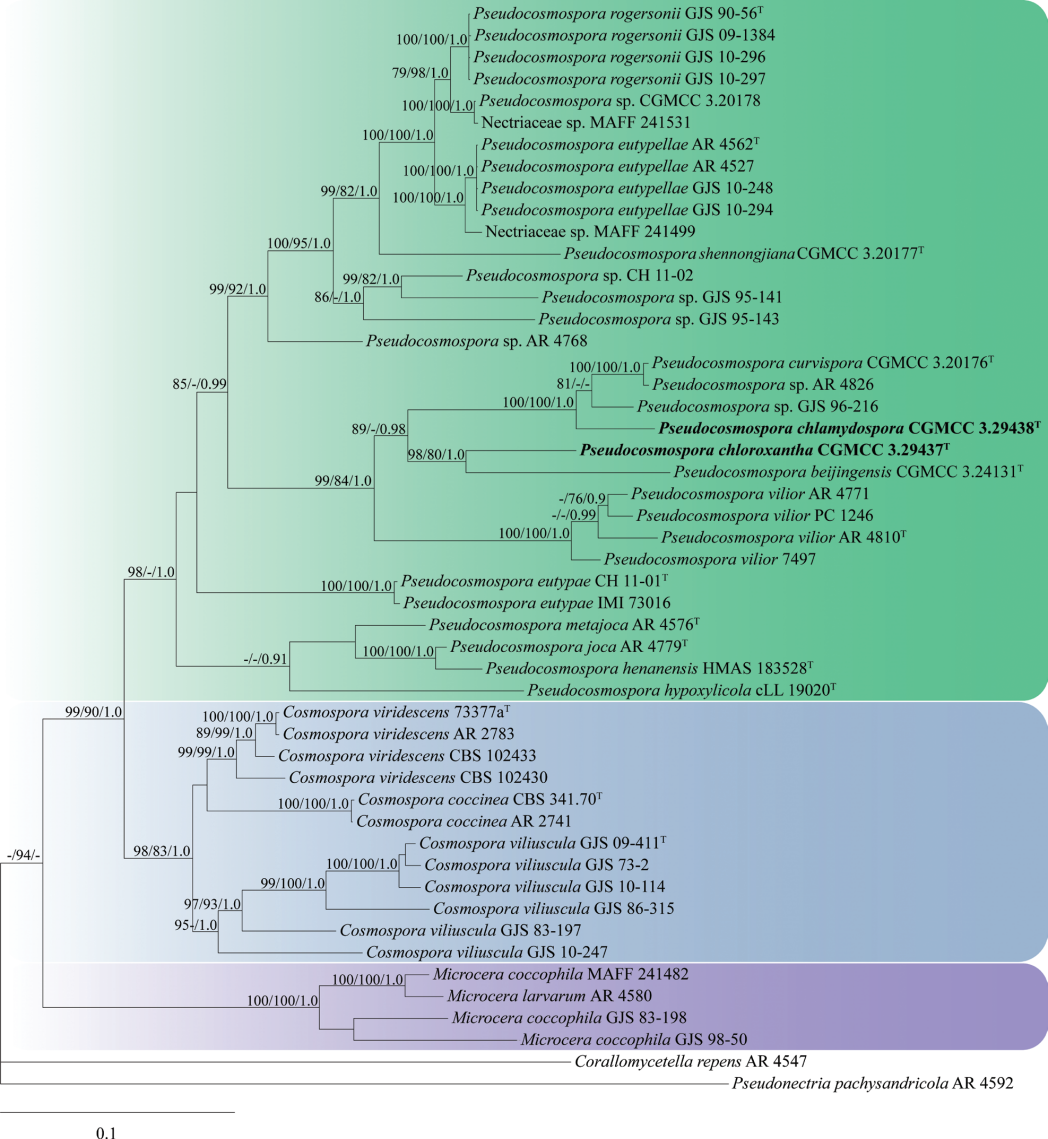
The Bayesian inference tree inferred from combined ITS, LSU, *tub2*, *rpb1* and *tef1-α* sequences of *Pseudocosmospora* species and other related species. MLBP (left) and MPBP (middle) (≥ 75%) and BIPP (right) (≥ 0.9) were shown at the nodes.

The sequences of ITS, LSU, *tub2*, *rpb1* and *tef1-α* from 50 strains of 29 species were analyzed. The concatenated sequence matrix was composed of 2,799 characters (ITS: 1–538, LSU: 539–1,323, *tub2*: 1,324–1,925, *rpb1*: 1,926–2,551, and *tef1-α*: 2,552–2,799) (Table 2). *Corallomycetella repens* (AR 4547) and *Pseudonectria pachysandricola* (AR 4592) were used as outgroup taxa. The BI tree was shown in Fig. 1. The topologies of the ML and MP trees were similar to that of the BI tree. The isolates CGMCC 3.29437 and 3.29438 were located among species of *Pseudocosmospora*, and received high statistical support values (MLBP/MPBP/BIPP = 98%/-/1.0). The isolate CGMCC 3.29437 grouped together with *P. beijingensis* (CGMCC 3.24131) (MLBP/MPBP/BIPP = 98%/80%/1.0), and the isolate CGMCC 3.29438 clustered with *P. curvispora* (CGMCC 3.20176), *Pseudocosmospora* sp. GJS 96-216 and AR 4826 (MLBP/MPBP/BIPP = 100%/100%/1.0). These six species formed a well-supported cluster (MLBP/BIPP = 89%/0.98), and were associated with *P. vilior* (MLBP/MPBP/BIPP = 99%/84%/1.0).

**Table 2.** Detailed characteristics of each sequence region.

Sequence region	No. of seq.	Length of alignment (bp)	No. of variable sites	No. of parsimony-informative sites
ITS	50	538	147	78
LSU	50	785	88	52
*tub2*	47	602	296	242
*rpb1*	46	626	298	245
*tef1-α*	41	248	223	196
ITS+ LSU+ *tub2*+ *rpb1*+ *tef1-α*	50	2,799	1,052	813

### Taxonomy

#### Pseudocosmospora chlamydospora

Taxon classificationFungiNectriaceae

Xiao Qian Wu, Z.Q. Zeng & W.Y. Zhuang sp. nov.

AAF66A27-4F56-589E-AC6B-DB21B8C1F5AF

Fungal Names: FN 573083

Figs 2, 3

##### Etymology.

The specific epithet refers to the chlamydospores produced in culture.

##### Typification.

China • Beijing City, Miyun District, roadside near Miyun Reservoir, on fruitbodies of *Paraeutypella karsti* growing on rotten bark, 8 September 2022, W.Y. Zhuang, Z.Q. Zeng & H.D. Zheng, 13067 (holotype HMAS 255842, ex-type culture CGMCC 3.29438).

##### Description.

***Mycelium*** not visible on natural substratum. ***Perithecia*** superficial, solitary to gregarious, non-stromatic or with a basal stroma, subglobose to globose with a truncate apex, laterally collapsed upon drying, orange-red to bright red, turning dark red in 3% KOH, becoming light yellow in 100% LA, 144–217 × 101–185 μm. ***Perithecial walls*** of two layers, 17–28 μm thick; the outer layer of textura angularis, 10–21 μm thick, cells 2.5–8 × 1.6–4.1 μm, walls 0.9–1.4 μm thick; the inner layer of textura prismatica, 5–10 μm thick, cells 4.7–13.2 × 1.6–3.3 μm, walls 0.8–1.3 μm thick. ***Asci*** clavate, with a simple apex, eight-spored, 42.7–56.7 × 3.4–5.7 μm. ***Ascospores*** ellipsoidal, 1-septate, hyaline to light yellow-brown, smooth-walled, uniseriate, overlapping obliquely, 5.9–8.8 × 3–4.7 μm.

**Figure 2.**
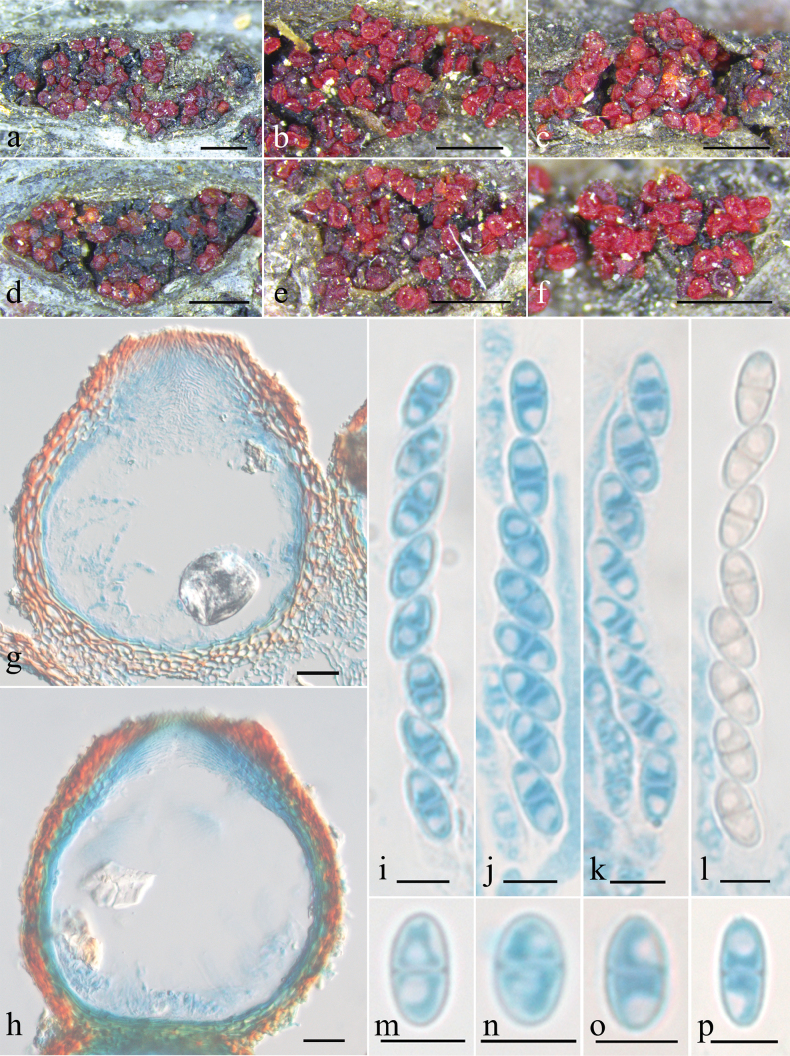
Macroscopic and microscopic features of *Pseudocosmospora chlamydospora* (HMAS 255842). **a–f**. Perithecia on natural substratum; **g, h**. Longitudinal section through perithecium; **i–l**. Asci with ascospores; **m–p**. Ascospore. Scale bars: 500 µm (**a–f**); 20 µm (**g, h**); 5 µm (**i–p**).

##### Colony characteristics.

***Colony*** on PDA 43 mm diam. after 28 d at 25 °C, crustose, beige, light yellow to salmon-pink, on SNA 44 mm in diam. after 28 d at 25 °C, with sparse whitish aerial mycelium. ***Conidiophores*** acremonium- to verticillium-like, septate, of indefinite length, hyaline, with 1–2 whorls and a terminal whorl of 2–4 phialides. ***Phialides*** subulate, tapering toward the apex, 5–24 μm long, 1.1–1.9 μm wide at the base, 0.6–1.2 μm wide at the tip. ***Conidia*** rod-shaped, fusiform, ellipsoidal, lemon-shaped or subglobose, rare clavate, unicellular, smooth, hyaline, non-guttulate, 2.5–5.7 × 1.3–2.7 μm. ***Chlamydospores*** globose to subglobose, rarely oblong, smooth-walled, hyaline, 3.4–8.4 μm in diam.

**Figure 3.**
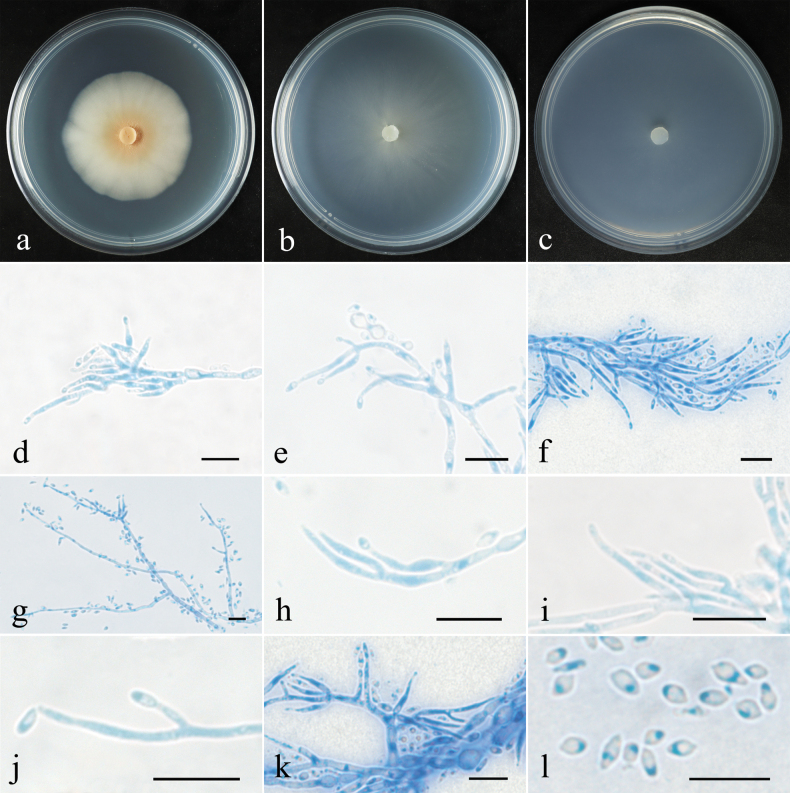
Colonial and microscopic features of *Pseudocosmospora chlamydospora* (CGMCC 3.29438). **a–c**. Colony after 28 d at 25 °C on PDA, CMD, SNA; **d–j**. Conidiophores and conidia; **k**. Conidiophores, chlamydospores and conidia; **l**. Conidia. Scale bars: 10 µm (**d–l**).

##### Notes.

Amongst the known species of *Pseudocosmospora*, *P. chlamydospora* most resembles *P. rogersonii* in similar host fungus, subglobose perithecia with a truncate apex, ellipsoidal and smooth ascospores, and pink colony on PDA (Herrera et al. 2013). However, the new species differs in having smaller asci (42.7–56.7 × 3.4–5.7 μm vs. 54–69 × 5.7–8.4 μm), shorter ascospores (5.9–8.8 μm vs. 7.9–12.2 μm), and producing globose to subglobose chlamydospores. Sequence comparisons revealed that there were 8 bp, 12 bp, 81 bp, 102 bp and 103 bp divergences detected for the ITS, LSU, *tub2*, *rpb1* and *tef1-α* regions between *P. chlamydospora* and *P. rogersonii* (Table 3). The multi-gene phylogenetic analyses showed that *P. chlamydospora* is closely related to *P. curvispora* and two unnamed species (MLBS/MPBP/BIPP = 100%/100%/1.0). However, *P. chlamydospora* can be easily distinguished by its shorter asci (42.7–56.7 µm vs. 53–68 µm) and ascospores (5.9–8.8 µm vs. 8–10 µm), wider conidia (1.3–2.7 µm vs. 0.8–1.2 µm), and forming globose to subglobose chlamydospores (Zeng and Zhuang 2021) (Table 4). Thus, both the morphological feature and sequence data indicated that they represent different taxa.

**Table 3.** Nucleotide sequence differences between the novel taxa and their close relatives.

Sequences region	ITS (bp)	LSU (bp)	*tub2* (bp)	*rpb1* (bp)	*tef1-α* (bp)
Comparing species					
*P. chlamydospora* vs. *P. rogersonii*	8	12	81	102	103
*P. chlamydospora* vs. *P. curvispora*	6	4	36	30	–
*P. chloroxantha* vs. *P. rogersonii*	15	4	81	98	101
*P. chloroxantha* vs. *P. chlamydospora*	10	8	70	82	84
*P. chloroxantha* vs. *P. beijingensis*	33	17	55	–	–

**Table 4.** Morphological comparisons of the new species and their closely related species.

Species	Perithecia size (µm)	Asci size (µm)	Ascospores size (µm)	Conidia shape	Conidia size (µm)	References
*P. beijingensis*	147–196 × 118–176	38–58 × 2.5–5	8–10 × 2.5–4	allantoid, curved	2.6–4.5 × 0.9–1.8	Zeng and Zhuang 2022
*P. chlamydospora*	144–217 × 101–185	42.7–56.7 × 3.4–5.7	5.9–8.8 × 3–4.7	rod-shaped, fusiform, ellipsoidal	2.5–5.7 × 1.3–2.7	This study
*P. chloroxantha*	132–230 × 127–210	53–76.8 × 4.7–9.2	7.1–11.5 × 3.7–6.3	rod-shaped to cylindrical	2.6–8.5 × 1.2–2.4	This study
*P. curvispora*	167–235 × 108–167	53–68 × 3–5	8–10 × 3–5	allantoid, mainly strongly curved	3–5 × 0.8–1.2	Zeng and Zhuang 2021
*P. rogersonii*	163–245 × 131–180	54–69 × 5.7–8.4	7.9–12.2 × 3.3–4.9	oblong to ellipsoidal	2.9–5.5 × 1.1–2.6	Herrera et al. 2013

#### Pseudocosmospora chloroxantha

Taxon classificationFungiNectriaceae

Xiao Qian Wu, Z.Q. Zeng & W.Y. Zhuang sp. nov.

F1C42434-0A94-5684-BD48-D3D82856505E

Fungal Names: FN 573084

Figs 4, 5

##### Etymology.

The specific epithet refers to the greenish yellow pigments on surface of colony.

##### Typification.

China • Beijing City, Yanqing District, Baili Landscape Gallery, on *Paraeutypella karsti* growing on rotten bark, 31 August 2022, W.Y. Zhuang, Z.Q. Zeng & H.D. Zheng, 13057 (holotype HMAS 255843, ex-type culture CGMCC 3.29437).

**Figure 4.**
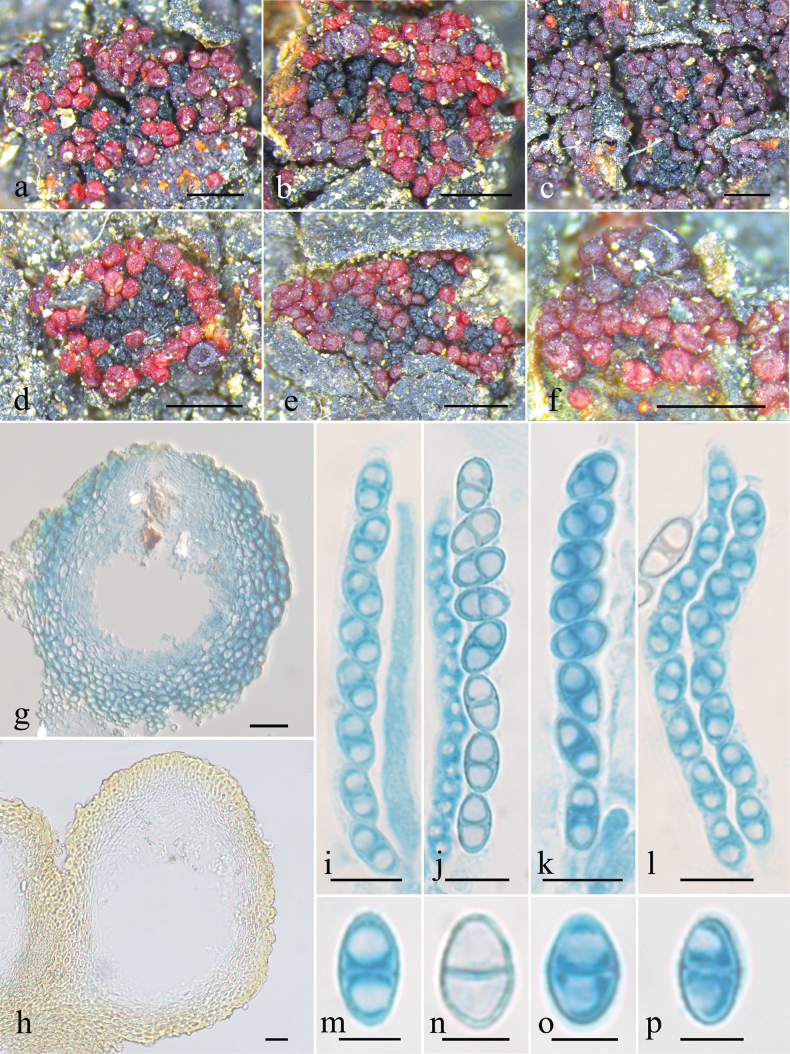
Macroscopic and microscopic features of *Pseudocosmospora chloroxantha* (HMAS 255843). **a–f**. Perithecia on natural substratum; **g, h**. Longitudinal section through perithecium; **i–l**. Asci with ascospores; **m–p**. Ascospore. Scale bars: 500 µm (**a–f**); 20 µm (**g, h**); 10 µm (**i–l**); 5 µm (**m–p**).

##### Description.

***Mycelium*** not visible on the natural substratum. ***Perithecia*** superficial and gregarious, non-stromatic or with a basal stroma, subglobose to globose with a truncate apex, laterally collapsed upon drying, bright red to brownish red, turning dark red in 3% KOH, becoming light yellow in 100% LA, 132–230 × 127–210 μm. ***Perithecial walls*** of two-layers, 19–37 μm thick; the outer layer of textura globulosa to t. angularis, 11–24 μm thick, cells 2.8–11.7 × 2–5.5 μm, walls 0.8–1.4 μm thick; the inner layer of textura prismatica, 9–15 μm thick, cells 5.9–14.5 × 1.6–3.1 μm, walls 0.8–1.7 μm thick. ***Asci*** cylindrical to clavate, with a simple apex or with an apical ring, eight-spored, 53–76.8 × 4.7–9.2 μm. ***Ascospores*** ellipsoidal to broad ellipsoidal, 1-septate, hyaline to light yellow-brown, smooth-walled, uniseriate, overlapping obliquely, 7.1–11.5 × 3.7–6.3 μm.

**Figure 5.**
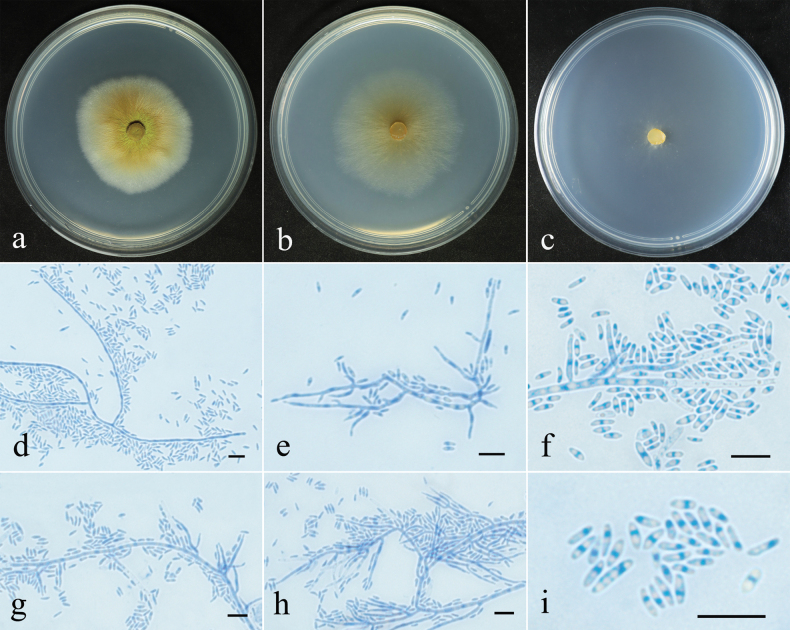
Colonial and microscopic features of *Pseudocosmospora chloroxantha* (CGMCC 3.29437). **a–c**. Colony after 28 d at 25 °C on PDA, CMD, SNA; **d–h**. Conidiophores and conidia; **i**. Conidia. Scale bars: 10 µm (**d–i**).

##### Colony characteristics.

***Colony*** on PDA 46 mm in diam. after 28 d at 25 °C, crustose, surface greenish yellow and reverse yellowish-brown, on SNA 42 mm in diam. after 28 d at 25 °C, with sparse whitish aerial mycelium. ***Conidiophores*** acremonium- to verticillium-like, septate, of indefinite length, and hyaline, with 1–2 whorls and a terminal whorl of 2–4 phialides. ***Phialides*** subulate, tapering toward the apex, 9.6–31.1 μm long, 0.9–2 μm wide at the base, 0.8–1.4 μm wide at the tip. ***Conidia*** rod-shaped to cylindrical, rarely ellipsoidal, unicellular, smooth-walled, hyaline, non-guttulate, 2.6–8.5 × 1.2–2.4 μm.

##### Notes.

Among the existing species of the genus, *P. chloroxantha* is morphologically similar to *P. rogersonii* and *P. chlamydospora* in having similar hosts, subglobose and laterally collapsed perithecia with a truncate apex, ellipsoidal and smooth ascospores, and conidia without guttules (Herrera et al. 2013). However, *P. chloroxantha* differs from *P. rogersonii* in longer conidia (2.6–8.5 μm vs. 2.9–5.5 μm) and rod-shaped to cylindrical conidia, and differs from *P. chlamydospora* in larger asci (53–76.8 × 4.7–9.2 μm vs. 42.7–56.7 × 3.4–5.7 μm), longer ascospores (7.1–11.5 μm vs. 5.9–8.8 μm), rod-shaped to cylindrical conidia, and the absence of chlamydospores. Additionally, there were 15 bp, 4 bp, 81 bp, 98 bp and 101 bp nucleotide divergences between the type of *P. chloroxantha* and *P. rogersonii*, and 10 bp, 8 bp, 70 bp, 82 bp and 84 bp unmatched loci were found between the type of *P. chloroxantha* and *P. chlamydospora* in the ITS, LSU, *tub2*, *rpb1* and *tef1-α* regions (Table 3). Phylogenetic analyses showed that *P. chloroxantha* is closely associated with *P. beijingensis* with strong support (MLBS/MPBP/BIPP = 98%/80%/1.0). However, the *P. chloroxantha* differs morphologically from the latter in having larger asci (53–76.8 × 4.7–9.2 µm vs. 38–58 × 2.5–5 μm), wider ascospores (3.7–6.3 µm vs. 2.5–4 µm), production of greenish yellow pigments in culture, and longer conidia (2.6–8.5 µm vs. 2.6–4.5 µm) (Table 4). Therefore, both morphological and molecular data support the establishment of *P. chloroxantha*.

## Discussion

In this study, *P. chlamydospora* sp. nov. and *P. chloroxantha* sp. nov. are described based on morphological characteristics and DNA sequence analyses of combined ITS, LSU, *rpb1*, *tub2* and *tef1-α* regions. Morphological observations revealed that they share key sexual- and asexual- features with other members of this genus, while a few differences existed that could set them apart. The result of phylogenetic analyses showed that with the addition of the two new species, the tree topology remains mainly consistent with the previous studies (Herrera et al. 2013; Zeng and Zhuang 2021). *Pseudocosmospora chlamydospora* and *P. chloroxantha* are closely related to, but clearly distinct from, the existing members of *Pseudocosmospora* (Fig. 1). Both morphological characteristics and muti-locus phylogenetic analyses support their placements in *Pseudocosmospora* and are new to science.

Both the morphology and DNA sequence analyses indicate the two new taxa have diatrypaceous hosts (Zhang et al. 2023), which are consistent with the other *Pseudocosmospora* species (Herrera et al. 2013). By parasitizing the fruitbodies of the hosts, and eventually suppressing or killing them, *Pseudocosmospora* species may be able to regulate the distribution of diatrypaceous and xylariaceous fungi in nature, thus maintaining the microbial community diversity and balance in forest ecosystems. Some Diatrypaceae species are reported as plant pathogens (Moyo et al. 2018), such as *Eutypa lata* and *Eutypella leprosa* are agents of diseases of grapevines (Munkvold et al. 1994; Díaz et al. 2011). Given the parasitic features, *Pseudocosmospora* species may have potential for biological control applications (Shiono et al. 2016). Moreover, some strains of *Pseudocosmospora* can produce metabolites with antimicrobial activity and cytotoxicity, which can inhibit plant pathogenic fungi and cancer cells, giving them great potential in agriculture and biomedicine (Hsiao et al. 2014; Shiono et al. 2016).

Since *Pseudocosmospora* was established by Herrera et al. (2013) who accepted ten species in the genus, several taxa were successively described (Zeng and Zhuang 2017, 2021, 2022; Lechat and Fournier 2020). Study of *Pseudocosmospora* in China was initiated by Teng (1934) who described *Nectria nummulariae* Teng [≡*P. nummulariae* (Teng) Z.Q. Zeng & W.Y. Zhuang] from Hainan Province. Additional ones were consecutively​ reported from different regions of the country (Zeng and Zhuang 2017, 2021, 2022). Until now, 14 *Pseudocosmospora* species are known in China, including the two newly described ones in this study. However, since the species diversity of the genus has not been fully revealed, further surveys in the unexplored regions with diverse vegetations are expected in the future.

## Supplementary Material

XML Treatment for Pseudocosmospora chlamydospora

XML Treatment for Pseudocosmospora chloroxantha

## Additional information

### Use of AI

No use of AI was reported.

### Author ORCIDs

Xiao-Qian Wu https://orcid.org/0009-0002-1662-7011

Wen-Ying Zhuang https://orcid.org/0000-0002-1162-9678

Zhao-Qing Zeng https://orcid.org/0000-0002-8460-7824

### Data availability

All of the data that support the findings of this study are available in the main text.
